# Swing-like pool boiling on nano-textured surfaces for microgravity applications related to cooling of high-power microelectronics

**DOI:** 10.1038/s41526-017-0014-z

**Published:** 2017-03-05

**Authors:** Sumit Sinha-Ray, Wenshuo Zhang, Barak Stoltz, Rakesh P. Sahu, Suman Sinha-Ray, Alexander L. Yarin

**Affiliations:** 1 Department of Mechanical and Industrial Engineering, University of Illinois at Chicago, Chicago, IL 60607-7022 USA; 2 Department of Mechanical Engineering, McMaster University, 1280 Main Street West, Hamilton, ON L8S 4L7 Canada; 3 Corporate Innovation Center, United States Gypsum, 700 US 45 N, Libertyville, IL 60048 USA; 4 Department of Materials Science and Engineering, Indian Institute of Technology, Indore, Madhya Pradesh 452017 India

## Abstract

Here, we demonstrate that heat removed in pool boiling from a heater mimicking high-power microelectronics could be used to facilitate a swing-like motion of the heater before being finally dissipated. This swing-like motion could be beneficial for shedding a large vapor bubble that encapsulates high-power heaters in microgravity where buoyancy force is unavailable for vapor bubble removal. The swing-like motion is propelled by vapor bubble recoil, the force which exists irrespective of gravity and buoyancy. We also demonstrate that this force could be significantly enhanced by depositing on the heater surface supersonically blown polymer nanofibers with cross-sectional diameters below 100 nm. These nanofibers provide additional nucleation sites, resulting in much more frequent bubble nucleation and departure, and thus a higher overall vapor recoil force propelling the heater motion. Such nanofibers strongly adhere to the heater surface and withstand prolonged harsh pool boiling. The measured velocity of the model swing-like heater in Novec 7300 fluid is about 1 cm/s.

## Cooling microelectronics with nanofibers

As microelectronics get smaller, there is an urgent need to develop efficient methods to keep them cool without extra power input. Under normal gravity, excess heat can be removed by vapor bubbles rising through a coolant. In space however, due to the lack buoyancy force, vapor bubbles remain attached to the submerged heater and prevent heat removal. Prof. Alexander Yarin, at the University of Illinois at Chicago, and his team show that in heaters mimicking high-power microelectronics, the thrust of vapor bubble release (the vapor recoil force, which exists irrespective of gravity) helps shedding merger vapor bubbles by generating a swing-like motion of the heater. Moreover, they demonstrate how nanofiber coatings can increase heat transfer by providing more bubble nucleation sites, and thus enhance the swing-like motion.

## Introduction

A recent thrive in miniaturization of circuit design in microelectronics increases a grave risk of failure of the onboard high-power microelectronics and computers. To enhance computation power manifold in miniature devices, a common trend is to pack more transistors in the integrated circuits (ICs). While modern ICs are already nearly saturated by the number of transistors, the current developments in lithographic processes promise ICs of about ~ 10 nm in size (http://www.nature.com/news/the-chips-are-down-for-moore-s-law-1.19338). That results in a tremendous Joule heating, thus making thermal management an acute problem, which can severely hinder development and operation of novel devices and even lead to failure due to the lack of an adequate thermal dissipation.^1–3^ Multiple cooling techniques have been explored so far, which include single-phase liquid cooling,^4, 5^ flow boiling,^6^ jet impingement cooling,^7, 8^ spray cooling,^9^ heat pipes,^10^ liquid metal cooling,^11^ targeted cooling with thermo-electric materials,^12^ and passive cooling employing phase change materials.^13, 14^ Pool boiling is another cooling method attractive for cooling of high-power mircoelectronics. Pool boiling benefits from significant latent heat of evaporation and could remove heat at the rate of ~ 100 W/cm^2^, although such direct/immersive type cooling is mostly limited to mainframe or supercomputer domain. Nucleate pool boiling regime can be realized on planar or wire- or fin-type heaters with microscopic roughness, selective wettability, porosity, etc.,^15–23^ with coolants such as deionized water, alcohols, Fluorinert fluids and fluid mixtures.^24–27^ Also, different suspensions^28–30^ and surfactant solutions^31^ have been used as coolants.

The critical heat flux (CHF) in pool boiling is reached when a practically intact vapor layer forms over the heater surface, which dramatically diminishes the heat transfer from heater to coolant, causing the heater to burn out.^32, 33^ Polymer nanofibers deposited on the heater surface not only enhance nucleate boiling but can also delay the onset of CHF. These can be electrospun nanofibers or supersonically solution-blown ultrafine nanofibers with cross-sectional diameters about 50 nm (ref. 34). Both types of the nanofibers can be either metalized,^35, 36^ or be used as pure polymer nanofibers.^37^ These nanofibers enhance nucleate boiling, significantly reduce the surface superheat, and thus enhance heat removal. It should be emphasized that the ultrafine supersonically solution-blown nanofibers strongly adhere to the heater surface, withstand prolonged boiling and provide an enormous number of active nucleation sites for vapor bubble formation.

In microgravity the buoyancy force, which removes vapor bubbles from the surface under normal gravity, is absent. As a result, vapor bubbles are not removed from the heater surface, but rather merge and form a large vapor bubble hovering over it and interrupting heat removal similarly to CHF (*cf*. Fig. 1 in SI). The efforts to prevent formation of the merger bubble in microgravity include subcooling and the application of the electric field.^38–40^ Subcooling facilitates continuous condensation on top of the large merger vapor bubble, which sustains nucleate boiling over the heater surface.^41^ The mechanical stirring was also employed.^42^ It should be emphasized that all these approaches involve an external source requiring power consumption, which is undesirable due to an extra energy dissipation. Moreover, the extra devices are also subjected to severe space and power restrictions characteristic of the microgravity applications.

**Fig. 1:**
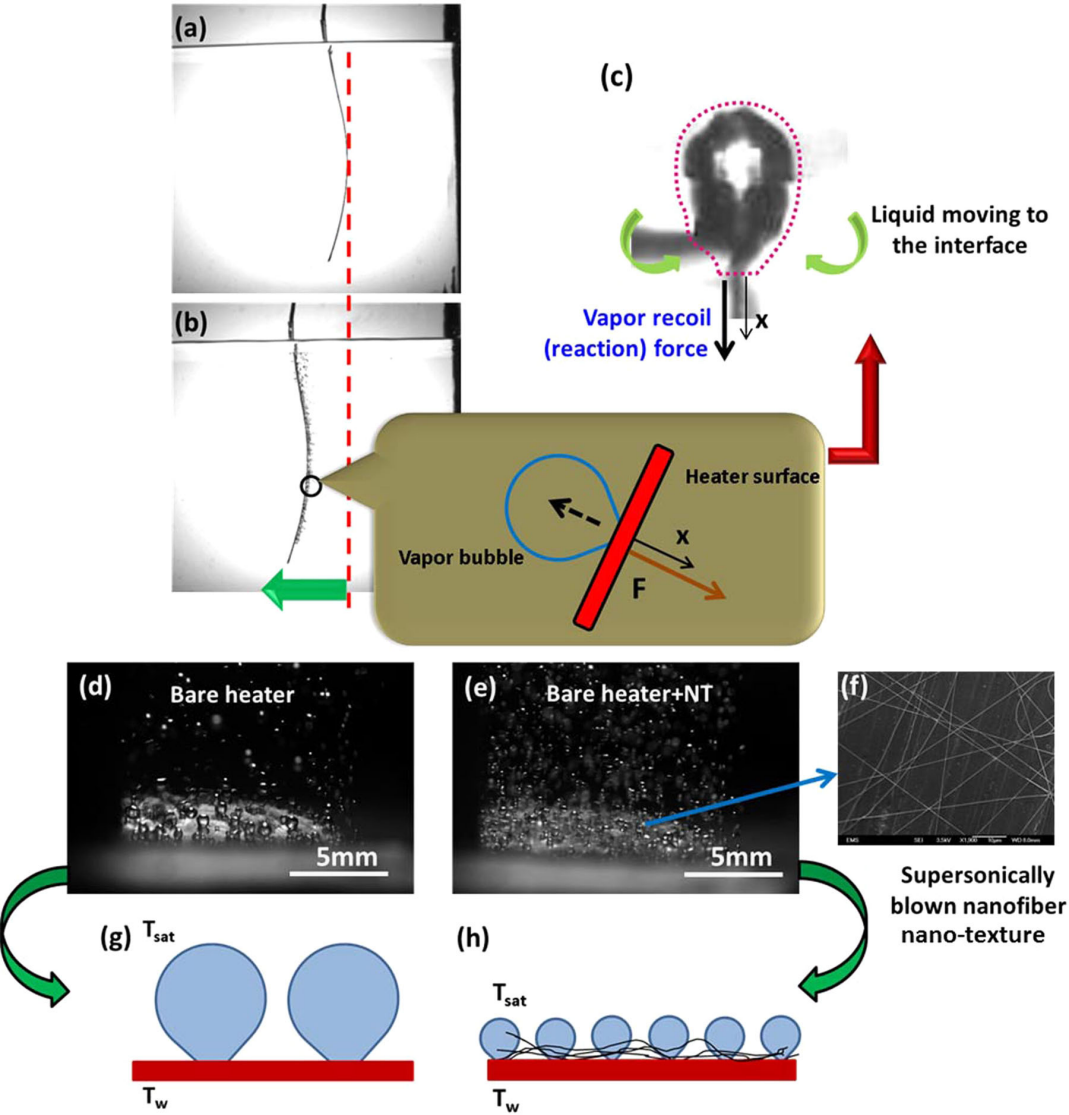
**a** Single Bare heater prior to boiling. **b** The heater displacement from the baseline (*red dashed line*) in 22 s after the inception of boiling. The *inset* shows a zoomed-in sketch of a single vapor bubble departing from the surface and the vapor recoil force shown by the bold arrow. **c** Photograph of a vapor bubble prior to the detachment with the boundary of the control volume surrounding its free surface and encompassing the heater surface under its footprint shown by the *dashed red line*. **d** Boiling on the Bare surface. **e** Boiling on the Bare+NT surface. **f** SEM image of Bare+NT surface. **g** and **h** Sketches of the characteristic sizes of vapor bubbles on the corresponding surfaces

Vapor recoil actuated by catalytic nano- or micromotor devices was in focus due to the efforts to mimic nano- or micro-scale biological organisms,^43–45^ albeit propulsion of macroscopic objects driven by vapor recoil was never studied, as to our knowledge. Here, we employ vapor recoil force resulting from the momentum balance and acting at a suspended heater surface. As a result, the heater surface is propelled from the emerging bubble and stirs the coolant in the pool. This mechanism employs a part of thermal energy removed from the heater surface to produce mechanical work before the ultimate dissipation, and does not require any additional power-consuming devices for mechanical stirring or electrical actuation. Moreover, vapor recoil thrust does not depend on the presence of gravity and the buoyancy force, and thus should be operational in microgravity. Here, we present the results of the ground experiments, which prove the concept.

## Results

### Single heater self-propulsion

Vapor-recoil-driven propulsion of single Kapton heaters was studied in two cases. In the first one, such a heater was used with no further modifications, i.e., its surface was bare. The corresponding results are denoted as Bare. In the second case, supersonically blown polymer nanofibers were deposited on the heater surface (*cf.* Fig. 1f), thus making it nano-textured. The corresponding results are denoted as Bare+NT. Figure 1a, b shows that when boiling begins at the convex side of the heater, it moves in the opposite direction due to the vapor recoil force. An arrow shows the direction of motion from the traced vertical dashed red line. Vapor bubbles leave the surface normally to it (Fig. 1c). In their further motion they are affected by the buoyancy force in the preset ground experiment. Irrespective of that, the heater surface has already moved away from the departed bubble, the mechanism which might be employed in microgravity.

Figure 1d, e shows two still images of nucleate boiling on the Bare and Bare+NT surfaces. It is seen that the Bare+NT surface facilitates an intense bubble nucleation, and the departing bubble sizes are smaller in comparison to those on the Bare surface (also see sketches in Fig. 1g, h), in agreement with our previous work.^37^

**Fig. 2:**
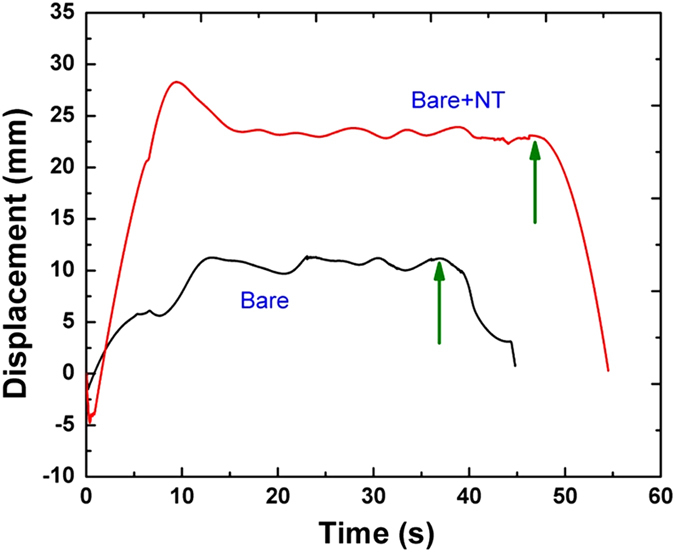
Displacement of a single heater. The *black line* corresponds to the Bare heater and the *red line*—to the Bare+NT heater. *Green arrows* denote the moments when the heaters were turned off

For both Bare and Bare+NT surfaces, heaters were propelled by vapor recoil force by several centimeters. The effect of vapor-recoil propulsion was enhanced by the presence of nanofibers (*cf.* Fig. 2), which is attributed to an enhanced bubble nucleation on nanofibers (*cf.* Fig. 1d with 1e). The intermediate plateau-like displacement achieved using the Bare+NT surface was by about 1 cm larger than that of the Bare surface. The Bare and the Bare+NT surfaces reached their plateau-like displacements in about 22 and 15 s, respectively, after the initiation of boiling. The resistance to a further motion is provided by the bending stiffness of lead wires used to suspend the heaters. As the heaters were turned off (marked by green arrows in Fig. 2), the “swing” started moving back due to the elastic forces in the suspending wires. It is worth mentioning that at the beginning of heating both the Bare and Bare+NT heaters slightly moved to the direction of the bubble departure, and then turned in the direction dictated by the vapor recoil force. This slight motion stems from the fact that at the beginning of boiling, the liquid in contact with the non-insulated side of the heater is warmer than in the bulk, and accordingly, the buoyancy force which is present in the ground experiments, can move the heaters counter to the vapor recoil force. This means that the vapor-recoil-driven propulsion observed in the present ground experiments is weakened by the buoyancy force. Still, as soon as vigorous boiling begins, the effect of the vapor recoil force significantly overbears the negative effect of buoyancy.

### Oscillatory motion of heater assembly

**Fig. 3:**
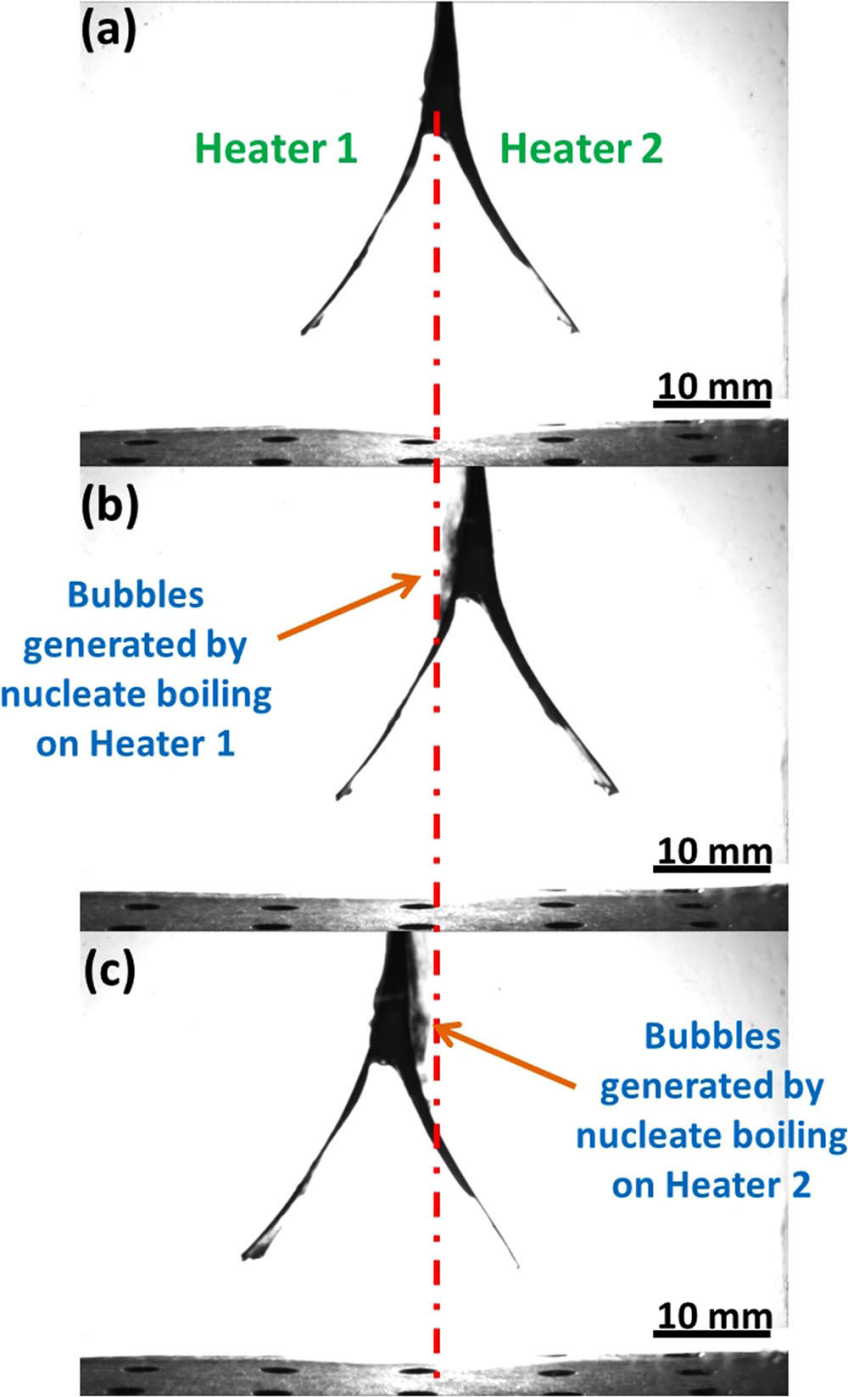
The alternating nucleate boiling on the concave sides of the two heaters in the assembly resulting in a swing-like motion driven by the alternating vapor recoil force. **a** The initial position. **b** Swing to the right, when the left-hand side heater (Heater 1) is operated, with the vapor recoil force propelling the assembly to the right. **c** Swing to the left, when the right-hand side heater (Heater 2) is operated, with the vapor recoil force propelling the assembly to the left

The assembly of two heaters (Fig. 3) was used to generate an oscillatory swing-like motion, aiming permanent stirring of the coolant by exploiting a part of the thermal energy removed from the heater as a work on the way to the complete dissipation. Each of the two heaters in the assembly mimics a high-power chip, and they are operated subsequently using a relay (*cf.* Fig. 2 in SI). The pulse generator (Fig. 2 in SI) activated the heaters in the assembly alternatingly and it was swinging back and forth being propelled by the vapor recoil force acting on each of them separately. In particular, the left-hand side heater (Heater 1 in Fig. 3) was operated for 6 s, then the right hand-side heater (Heater 2 in Fig. 3) was operated for 5 s, etc. The suspending lead wires were rather stiff and introduced an unevenness due to the bending stiffness resisting the heater motion, which was compensated by the unequal operational times used. This guaranteed the swing-like motions with almost equal amplitudes in both directions, as illustrated by the snapshots from a high-speed video in Fig. 3. The heaters were operated by applying the voltage of 115 V. The end-to-end distance between the heater ends at the base of the assembly ‘‘triangle’’ was 25 mm and it did not change during the experiments due to the rigidity of the heater assembly. In the experiments, 7.1 cm of the heater length was initially submerged in the coolant, while the top 0.52 cm was above the free surface. The horizontal and vertical coordinates X and Y of the center of the initially submerged part (*cf.* Fig. 3 in SI) were measured as functions of time from the high-speed video using MATLAB.

**Fig. 4:**
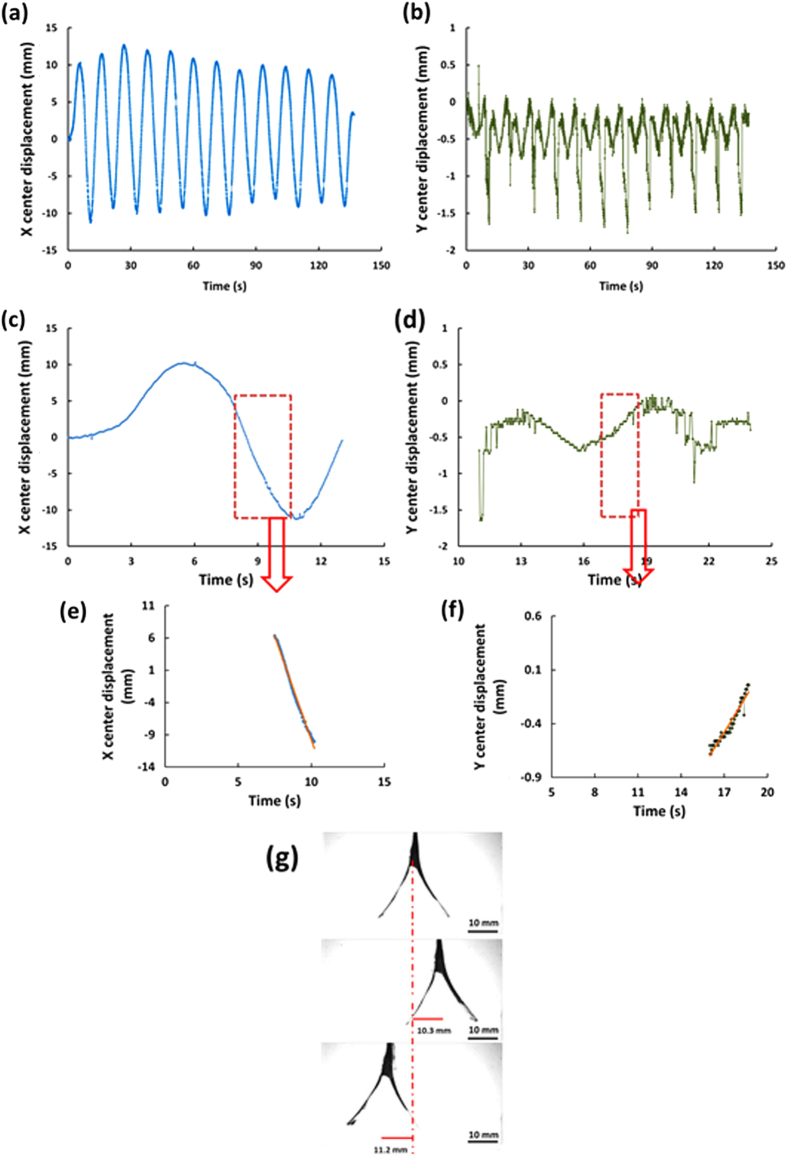
Swing-like motion of the assembly of two Bare heaters. **a** The displacement of the center in the X-direction vs. time. **b** The displacement of the center in the Y-direction vs. time. **c** The X displacement of the heater assembly for the first 14 s. **d** The Y displacement of the heater assembly for 11–25 s. **e** An almost linear section of the dependence X(t), with the *blue line* being the data and the *orange line*-the linear approximation, where the velocity |d*X*/d*t*| = 0.64 cm/s. **f** An approximately linear section of the dependence Y(t), with the *green line* being the data and the *orange line*-the linear approximation, where the velocity |d*Y*/d*t*| = 0.22 cm/s. **g** Several snapshots of the heater assembly motion at the peak amplitudes being 10.3 mm—for Heater 1 and 11.2 mm—for Heater 2

The overall swinging time in the experiments was 140 s, and the recorded time series X(t) and Y(t) for the Bare heater assembly are shown in Fig. 4a, b, respectively. It should be emphasized that at the beginning (for the first two cycles), the maximal displacement amplitude X(t) increased as a function of time, while later on it slightly diminishes. This was in accord with the temperature drop of the bulk fluid in time (*cf.* Fig. 4 in SI). The decrease in the temperature of the bulk fluid was in part due to the stirring provided by the swing-like motion itself! The velocity components |d*X*/d*t*| and |d*Y*/d*t*| of the swing-like motion were found from the linear sections of the times series shown in Fig. 4c, d. The measured values of |d*X*/d*t*| and |d*Y*/d*t*| were 0.64 and 0.22 cm/s, respectively, (*cf.* Fig. 4e, 4f). The velocity magnitude of the heater assembly [(d*X*/d*t*)^2^+(d*Y*/d*t*)^2^]^1/2^ was found as 0.68 cm/s. Also, Fig. 4g shows the snapshots from the high-speed video of the heater assembly motion corresponding to the peak amplitudes of the swing-like motion.

**Fig. 5:**
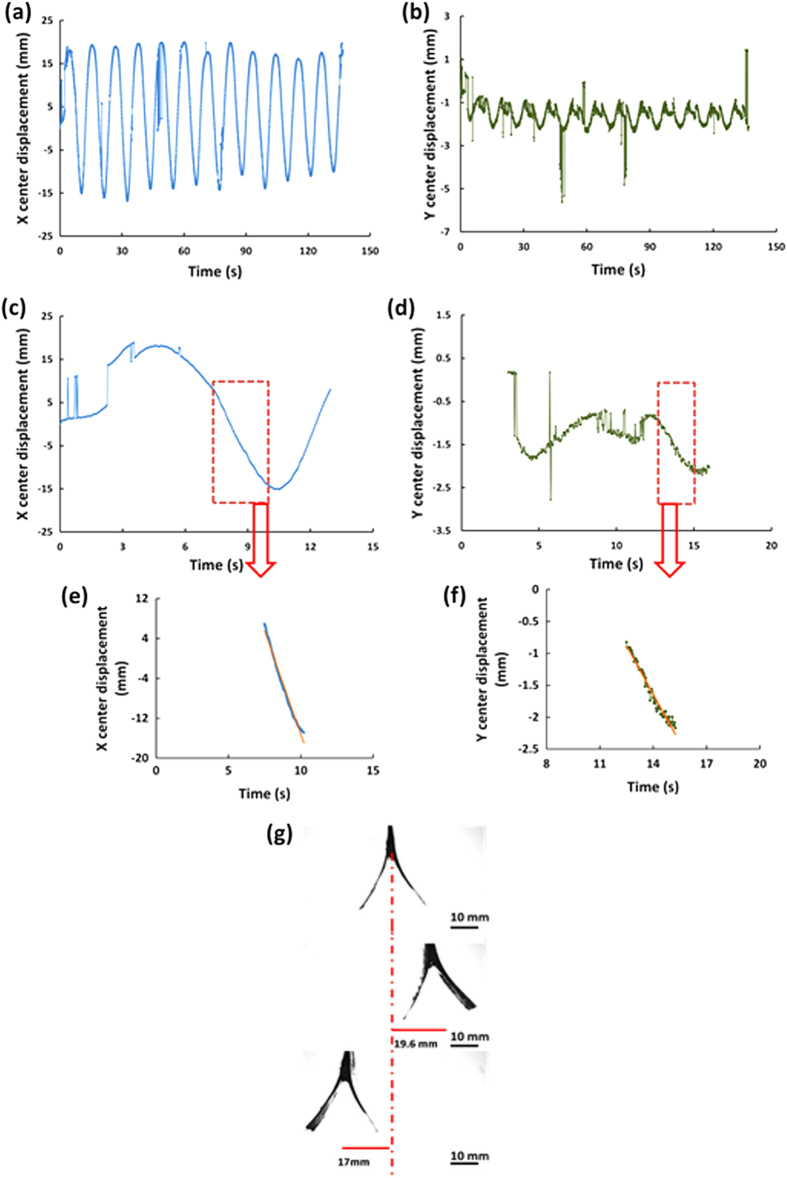
Swing-like motion of the assembly of two Bare+NT heaters. **a** The displacement of the center in the X-direction vs. time. **b** The displacement of the center in the Y-direction vs. time. **c** The X displacement of the heater assembly for the first 14 s. **d** The Y displacement of the heater assembly for 4–16 s. **e** An approximately linear section of the dependence X(t), with the *blue line* being the data and the *orange line*-the linear approximation, where the velocity |d*X*/d*t*| = 0.83 cm/s. **f** An approximately linear section of the dependence Y(t), with the *green line* being the data and the *orange line*-the linear approximation, where the velocity |d*Y*/d*t*| = 0.50 cm/s. **g** Several snapshots of the heater assembly motion at the peak amplitudes being 19.6 mm—for Heater 1and 17 mm—for Heater 2

The presence of the supersonically blown polymer nanofibers on the heater surfaces (the Bare+NT heater assembly) facilitates bubble nucleation (*cf.* the previous section and ref. 37) and increases the vapor recoil force. This results in larger swing amplitudes and an increase in the velocity component |d*X*/d*t*| up to 0.83 cm/s, i.e., a 29% enhancement compared to the Bare heater (*cf.* Fig. 5a–5g). The corresponding velocity component |d*Y*/d*t*| was found as 0.50 cm/s, a 127% increase compared to the Bare heater. Accordingly, the velocity magnitude increased to 0.97 cm/s, i.e., by 39% compared to the Bare heater.

### Nucleation of a single bubble

**Fig. 6:**
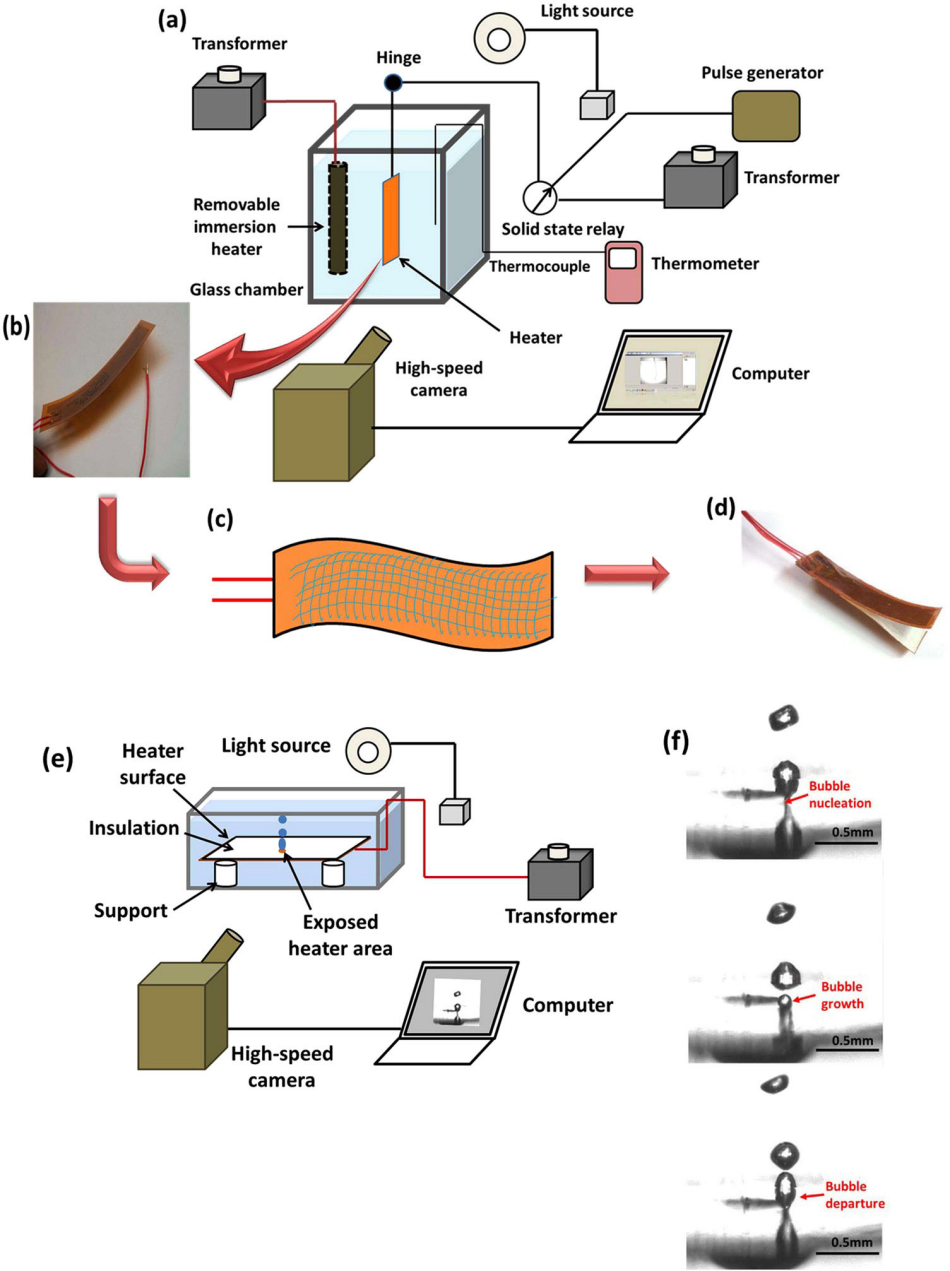
Experimental set-up and the flexible heater surface. **a** Pool boiling cell **b** Photograph of the heater. **c** Sketch of a heater with deposited nanofiber mat. **d** Photograph of the heater assembly with two convex sides being attached after a proper insulation, and boiling occurring only on the outside concave sides. **e** Single bubble nucleation cell. **f** Three snapshots elucidating a single bubble nucleation, growth, and detachment from a single nucleation site. The total time span is 9.3 ms

Nucleation of a single bubble from a single nucleation site in a specially designed cell was studied in detail to reveal the bubble nucleation and growth and elucidate its departure from the heater surface (Fig. 6e, f). The time span between the bubble nucleation and departure was about 9.32 ms. The height of the bubble at the moment of departure was about 0.4 mm. The top surface of the bubble moved with the acceleration of about 2.3 m/s^2^.

## Discussion

As a vapor bubble nucleates and grows, it reaches a critical size either determined by the surface imperfections on the Bare surfaces or by the inter-fiber distance in the case of the Bare+NT surfaces.^37^ Then, the bubble detaches from the surface providing the vapor recoil force, which is the thrust required for heater propulsion. The vapor recoil force is directed along the x-axis normal to the heater surface (*cf.* Fig. 1c). The momentum balance for the control volume that encompasses the bubble surface and the heater part under the bubble footprint, reveals the two components, which provide the vapor recoil (the reaction force, *cf.* Fig. 1c) acting on the vertical heater surface. Namely, (i) *ρ*_v_ *u*_v_^2^ *S*, and (ii) *m*_v_ *a*, where *ρ*_v_ is vapor density, *u*_v_ is the average vapor velocity, *S* is the footprint area, *S* ≈ π*R* ^2^, with *R* being the characteristic bubble radius, *m*_v_ ≈ 4*ρ*_v_π*R* ^3^/3 is the vapor mass inside the bubble, and *a* = d*u*_v_/d*t* being the vapor acceleration (*t* is time). Accordingly, the vapor recoil force is expressed as *F*_x_ = *ρ*_v_ *u*_v_^2^ *S*+*m*_v_ *a*. Note, that the expression for the vapor recoil force *F*_x_ = *η* ^2^(*ρ*_v_^−1^−*ρ*_L_^−1^)*S* used in ref. 46, with *η* = *ρ*_v_ *u*_v_ being the evaporation rate per unit surface area, and *ρ*_L_ being the liquid density, implies *a* = 0, and the liquid supply through the surface, which is not the case near a solid surface where liquid is supplied parallel to the surface, as is shown in Fig. 1c by the green arrows. A steady-state motion of the heater implies that *F*_x_ = *F*_D_, where the drag force imposed on the moving heater by the surrounding liquid is equal to *F*_D_ = *C*_D_ *ρ*_L_ *V* ^2^ *S*/2, where *C*_D_ is the drag coefficient, and *V* is the heater velocity.^47, 48^ Accordingly, *V* = [(2/*C*_D_)(*ρ*_v_/*ρ*_L_)(4*R*a/3+*u*_v_^2^)^1/2^], which is also applicable to any surface with dense nucleation and bubble detachment.

Evaluate the vapor velocity *u*_v_ = *η*/*ρ*_v_ using the fact that the evaporation rate *η* = *q*/*L*, with *q* being the heat flux supplied to the heater, and *L* being the latent heat of evaporation. In the present case *q* = 15.5 kW/m^2^ and *L* = 101.74 kJ/kg, which yields *η* = 0.152 kg/(m^2^ × s). The vapor density is evaluated from the ideal gas law as *ρ*_v_ = *PM*_w_/*R*_g_ *T*, where *P* is the vapor pressure [approximately, the atmospheric pressure of 101.172 kPa], *M*_w_ is the molecular weight of Novec 7300 [0.35 kg/mol], *R*_g_ is the universal gas constant, and *T* is the boiling temperature of 371 °K. Accordingly, *ρ*_v_ = 11.5 kg/m^3^. Then, *u*_v_ = 0.0132 m/s. The liquid density is *ρ*_L_ = (1.7162−0.0024T) g/mL (here T is in °C) (http://multimedia.3m.com/mws/media/338713O/3mtm-novectm-7300-engineered-fluid.pdf), which yields *ρ*_L_ = 1.48 g/cm^3^ or 1480 kg/m^3^.

Taking for the estimate *R* = 2 × 10^−4^ m and *C*_D_ ≈ 1.28 (ref. 47), we obtain *V* = 0.231 cm/s, which is of the same order as the heater velocities measured experimentally.

The detaching bubbles are larger in the case of the Bare surfaces and smaller in the case of the Bare+NT surfaces (*cf.* Fig. 1d, e). Also, the bubble nucleation and detachment rate is much higher on the Bare+NT surfaces compared to the Bare surfaces. These factors facilitate faster motion of the Bare+NT surfaces in comparison with the Bare surfaces.

It should be emphasized that supersonically blown ultrafine polymer nanofibers (below 100 nm in cross-section) strongly adhere to the underlying heater surface due to the van der Waals forces,^37^ which allows such coatings to withstand vigorous pool boiling even in water for many hours. Therefore, their presence has a long-lasting effect on the swing-like motion, facilitating it, which in turn, facilitates the overall stirring of coolant in the pool. As a result, the coolant bulk temperature decreases in time (*cf.* Fig. 4 in SI), which further facilitates a decrease in the surface superheat. Moreover, in microgravity such an intense mixing (sustained by a part of the energy removed from the heater and requiring no extra energy supply) would prevent merger bubble formation near the heater surface and thus delay the onset of CHF associated with the absence of the buoyancy force.

It should be emphasized that the initial vertical orientation of the heater in the experimental set-up makes the buoyancy force and the vapor recoil force orthogonal to each other. This completely eliminates the buoyancy effect on the initiation of the swing-like motion of a heater with the velocity of ~ 1 cm/s. In microgravity where the buoyancy force is practically absent, the situation is exactly the same, and thus irrespective of the presence or absence of the gravity force, the swing-like motion of the heater with an asymmetric bubble detachment, as in the present case, can be triggered solely by the vapor recoil force. Moreover, under the normal gravity conditions the bubbles detached normally to the heater surface would rise due to buoyancy. Since the continuity equation requires filling voids, fluid flow arises under the normal gravity conditions from the frontal side of the heater toward the rear side where the bubbles are formed, i.e., in the direction opposite to the heater motion. As a result, due to the buoyancy effect, an additional drag acts on swinging heaters under the normal gravity conditions. Nevertheless, the thrust associated with vapor recoil force is sufficient to overbear even this extra drag under the normal gravity conditions, as the present results clearly demonstrate.

In summary, the vapor recoil force acts on the bare heater surfaces, as well as on the heater surfaces covered with ultrafine nanofibers. The vapor recoil force facilitates self-propulsion of the heater, which essentially runs away from vapor bubbles released from its surface. That is the main mechanism, which prevents formation of screening macro-bubble near the heater surface due to merging of any ‘‘un-lifted’’ micro-bubbles. Since vapor recoil force exists in microgravity, heater self-propulsion would manifest itself in microgravity as well. Accordingly, macro-bubble formation would be avoided and the operation of heat transfer system based on such self-propelling heaters sustained. This mechanism exists without any ultrafine nanofibers on the heater surface, albeit it can be enhanced by them.

## Materials and methods

### Materials

Kapton-insulated flexible heater with dimensions of 7.62 × 2.54 cm and thickness of 0.0254 cm were purchased from Omega. The heaters were rated for 115 V and 1.55 W/cm^2^. Novec 7300 fluid, obtained from 3 M, was used as the working fluid. Apart from the Bare heater surfaces, polyacrylonitrile (PAN) nanofiber-coated heater surfaces were also used in the present experiments. PAN (*M*_w_ = 200 kDa) and its solvent N, N-dimethylformamide, were both purchased from Sigma-Aldrich.

### Experimental system: design

The experimental scheme is depicted in Fig. 6, which shows two set-ups for two different experiments. The first set-up (Fig. 6a–6d) was used to observe the swing-like motion, and the second one (Fig. 6e–6f)—to observe nucleation and departure of a single bubble from a heater. The first set-up (*cf.* Fig. 6a) consists of a glass chamber containing working fluid (Novec 7300), the heater (or the heater assembly), two transformers, a relay switch operated by a pulse generator, and a TC-08 thermocouple data acquisition module (from Omega). The glass chamber is lined with aluminum panels, with aluminum bottom and the top being open. Two different types of heater surfaces were used in the pool boiling experiments. The high-power heater used in these experiments was a flexible Kapton heater submerged in the coolant (Novec 7300 fluid) pool. The pool was heated up to 96 °C by an immersion heater (see Fig. 6a). The immersion heater was removed as soon as this temperature was reached. The heating element of the flexible heater is an etched foil of nichrome sandwiched between two polyimide films with fluorinated ethylene propylene adhesive. The nichrome and polyamide have different thermal expansion coefficients (14 × 10^−6^ K^−1^ for nichrome vs. 60 × 10^−6^ K^−1^ for polyimide). As a result, the Kapton heater has a tendency to buckle if heated in air, as is seen in Fig. 6b. No additional buckling/deformation of the heater due to the mismatch in the thermal expansion coefficients was observed when it was submerged in the coolant.

One side of a Kapton single heater was insulated with Recollection insulator paper and boiling occurred on the concave side when power was supplied. This single-side boiling resulted in the vapor recoil force, which propelled the heater motion. Bubble nucleation was enhanced when the non-insulated side of the heater (7.1 × 2.54 cm) was covered by a thin net of supersonically blown ultrafine polymer nanofibers, as sketched in Fig. 6c and shown in Fig. 1f.

To form heater assemblies used in the experiments with the swing-like motion, two heaters were glued together (using the silicon adhesive RTV Sealant 732) on top of their insulated convex sides (*cf.* Fig. 6d). Then, nucleate boiling occurred on the outside concave heater surfaces. Boiling was actuated subsequently on each side of the heater assembly.

Before the single flexible heaters or the heater assemblies were operated, an immersion heater (Omega) was employed to preheat the liquid to 96 °C. A T-type thermocouple was inserted in the bulk liquid to monitor the temperature, but not to interfere with the heaters motion. As soon as the temperature had reached 96 °C, the immersion heater was removed and a single heater or a heater assembly was inserted in the liquid. The heaters were suspended on a mechanical hinge through a suspension lead wire of 25.4 cm in length. The upper 0.52 cm part of the heater length was left in air, while the lower 7.1 cm part was submerged in the liquid. All the heaters were connected to a variable auto-transformer (120 V AC) operating at 115 V, which alternatingly powered the heaters through a solid-state relay. The two sides of the heater assemblies were operated alternatingly during certain periods determined by a signal/pulse generator (Berkeley Nucleonics Corporation Model 555 Pulse generator). The simplified circuit diagram is shown as Fig. 2 in SI. Heater 1 (on the left-hand side in Fig. 3) was operated for 6 s, and Heater 2 (on the right-hand side in Fig. 3) was operated for 5 s.

This periodic switching of the heaters in the heater assembly resulted in a swing-like motion of the assembly in liquid. The entire process was recorded using a high-speed camera Phantom V210 at 24 fps, using a light emitting diode light source for proper illumination. From the beginning of the process to the end of recording the bulk liquid temperature decreased from 95.39 °C (±0.12 °C) to 94.8 °C (±0.11 °C), which was facilitated by stirring of the liquid by the swinging heater assembly, which in turn, slightly diminished the swinging amplitude in time. In all the experiments, 800 mL of Novec 7300 engineered fluid were used. The experimental set-up did not have an enclosure chamber, and that is why the liquid was raised to a temperature below the saturation point, being warm enough to easily trigger nucleate boiling on the surfaces of the flexible heaters, when they were powered.

To quantify heater motion, the video recordings were analyzed using MATLAB. In the case of a single heater, the bottom part was tracked and the distance measured was calibrated with a known length of 7 mm. In the case of the heater assembly, the lower end-to-end distance of 25 mm was used for calibration. The motion of the bottom-center point was measured from the video (*cf.* Fig. 3 in SI).

The visualization of nucleation, growth, and departure of a single bubble was conducted as shown in Fig. 6e, f. A single heater was sandwiched between two insulator papers. A hole of 0.31 mm was left open on the insulator paper facing the liquid to expose the heater surface to it. The bubble nucleation, growth, and departure were recorded using the above-mentioned high-speed camera at 28311 fps. Image-Pro Plus 6.0 (Media Cybernetics) was used for measurements involving bubble nucleation, growth, and departure and the still images extracted from the high-speed video were analyzed. The calibration of 0.17 mm was used for the measurements.

### Supersonic solution blowing

Supersonic solution blowing was conducted following the previous work of the present group,^34^ as depicted in Fig. 5 in SI. In brief, the 4 wt% PAN solution was supplied through a 25 G needle using a syringe pump (New Era Pump Systems Inc.) at a flow rate of 0.4 mL/h. The needle was connected to a DC high-voltage source, while the nozzle (the Laval nozzle, Silvent 209-L) was grounded. The supplied voltage was 6.4 kV and the distance between the needle and the nozzle was 1 cm horizontally and 2.54 cm vertically. The supply air pressure was 70 psi and the air velocity at the Laval nozzle exit was previously measured^34^ as 564 m/s. The polymer jet issued from the needle, was electrified and, accordingly, attracted to the Laval nozzle. On the way to the nozzle it underwent the electrically driven bending instability^49^ and had already become very thin. After that, near the Laval nozzle, it was swept by the supersonic air flow and stretched by it (directly, and via the aerodynamically driven bending instability).^49^ At all stages, the solvent evaporated and, accordingly, the solidified nanofibers deposited on the heater surface were about ~ 100 nm in cross-section. A predetermined part of a heater surface was kept unmasked, i.e., open for the nanofibers deposition. Essentially, the heater was used as a substrate during the deposition process and kept at a distance of 30 cm from the exit of the Laval nozzle. The rest of the heater surface was masked using paper-tape (3 M). After the nanofiber deposition had been completed, the tapes were removed without causing any delamination of the nanofiber layers. The deposition process continued for 2 min. Afterwards the heaters were stored and used in the boiling experiments without any further modification.

## Electronic supplementary material

Supplementary Information
