# Supplementary material for: Swing-like pool boiling on nano-textured surfaces for microgravity applications related to cooling of high-power microelectronics
Source: NPJ Microgravity. 2017 Mar 5;3:9. doi: 10.1038/s41526-017-0014-z (PMC5460202; doi:10.1038/s41526-017-0014-z)
Supplement: Supplementary file 1 — Supplementary Information [file 41526_2017_14_MOESM1_ESM.docx]

**Supplementary Information**

**Swing-like Pool Boiling on Nano-textured Surfaces for Microgravity Applications Related to Cooling of High-Power Microelectronics**

Sumit Sinha-Ray^1^, Wenshuo Zhang^1^, Barak Stoltz^1^, Rakesh P. Sahu^1,2^, Suman Sinha-Ray^1,3,4^, Alexander L. Yarin^1*^

^1^ Department of Mechanical and Industrial Engineering, University of Illinois at Chicago, Chicago, Illinois 60607-7022, USA

^2^ Department of Mechanical Engineering, McMaster University, 1280 Main Street West, Hamilton, Ontario L8S 4L7, Canada

^3^ Corporate Innovation Center, United States Gypsum, 700 US 45 N, Libertyville, IL-60048, USA

^4^ Department of Materials Science and Engineering, Indian Institute of Technology, Indore, Madhya Pradesh 452017, India


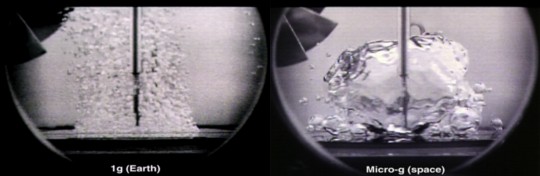


**Figure 1.** Pool boiling at normal gravity (on the left-hand side panel) and in microgravity (on the right-hand side panel)^1^.

***
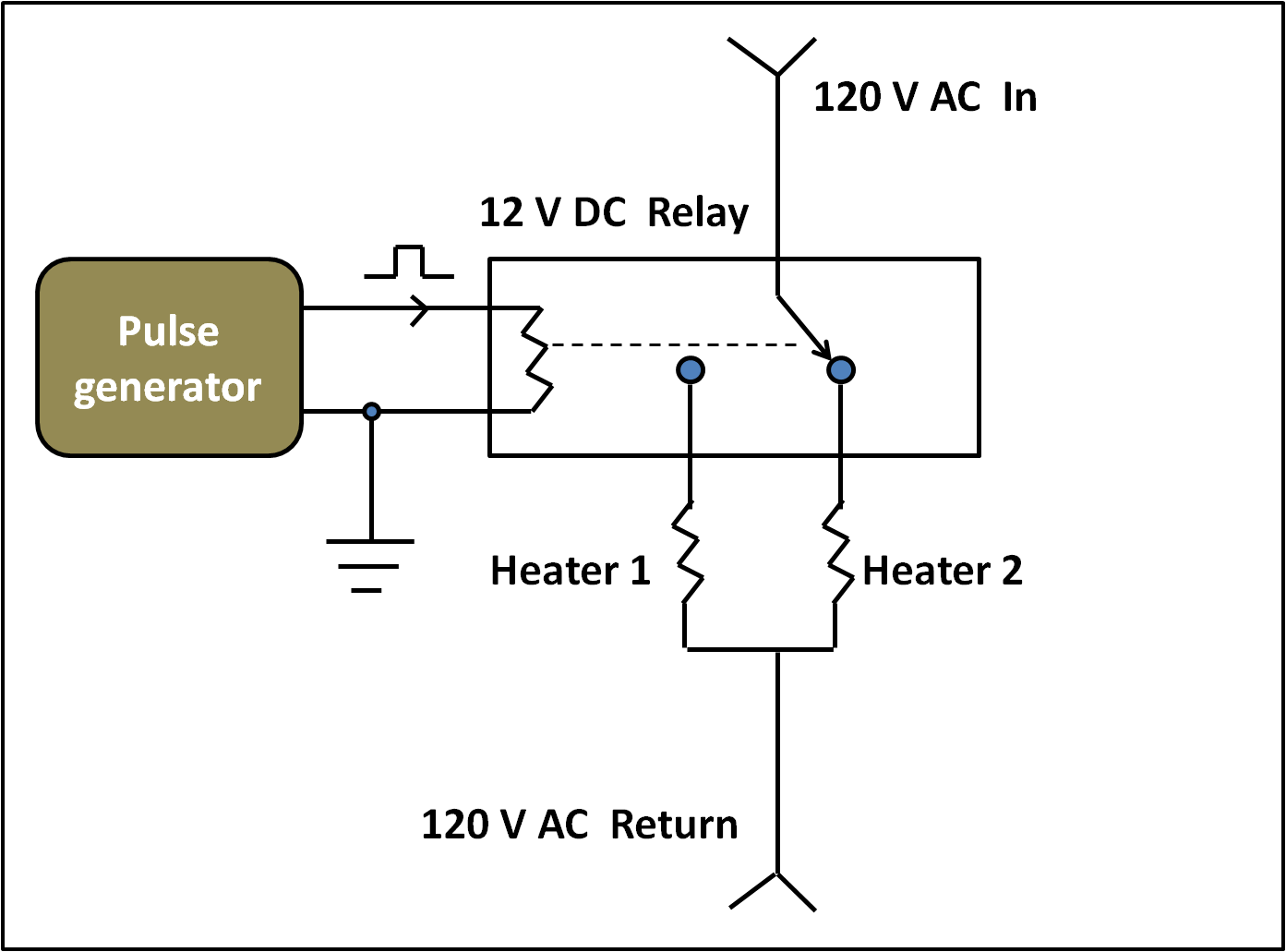
***

**Figure 2:** Circuit design for the heater assembly with a solid-state relay and the pulse generator.


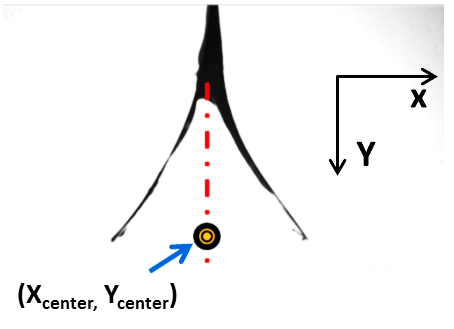


**Figure 3:** Center point of the heater assembly.


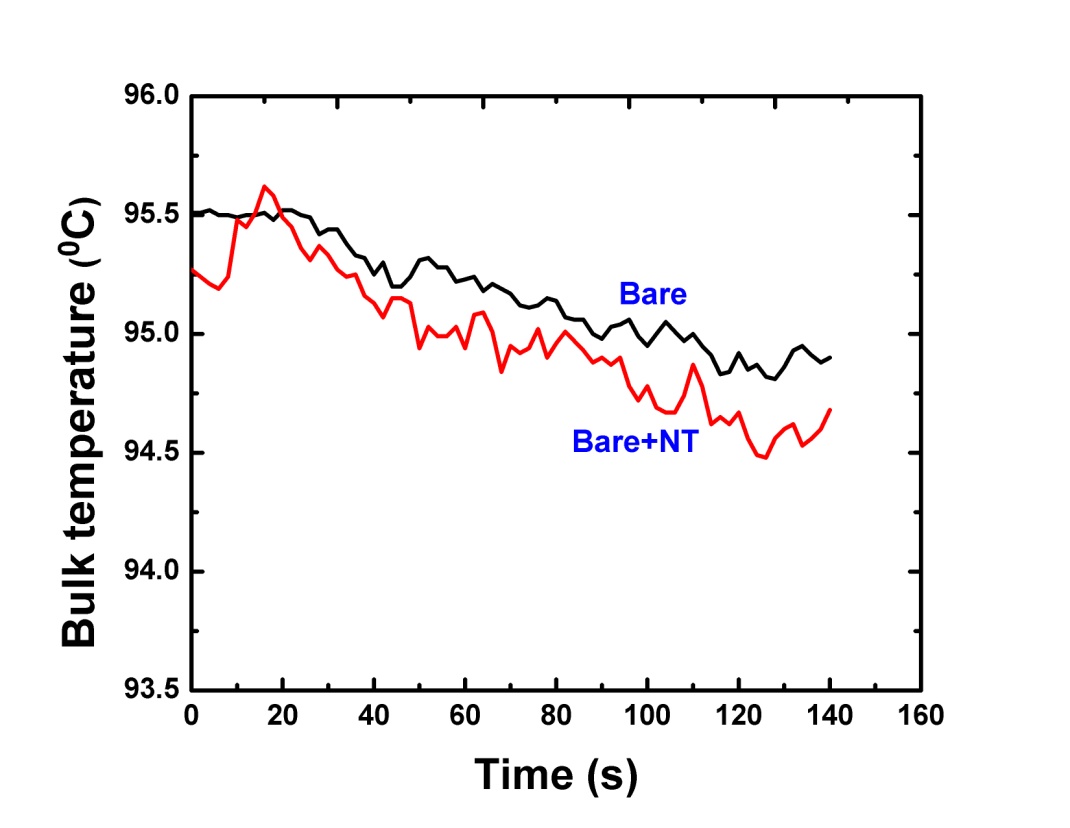


**Figure 4:** Temperature of the bulk liquid during the swing-like motion of the heater assembly. A thermocouple was inserted in the liquid and did not move.


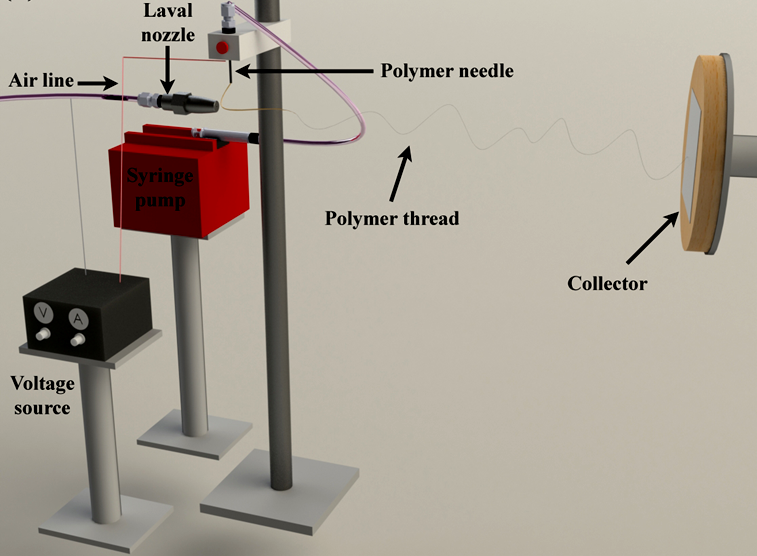


**Figure 5:** Schematic of the supersonic solution blowing and nanofiber deposition on the collector.

REFERENCES

1. <http://science.nasa.gov/science-news/science-at-nasa/2001/ast07sep_2>.
